# Case report: right-sided native endocarditis presenting with multiple abscess formations [care compliant]

**DOI:** 10.1097/MD.0000000000015961

**Published:** 2019-06-14

**Authors:** Tsung-Yu Chan, Chih-Chia Hsieh, Chien-Liang Chen, Yao-Yi Huang, Chia-Chang Chuang

**Affiliations:** a Department of Emergency Medicine, National Cheng Kung University Hospital, College of Medicine, National Cheng Kung University, Tainan; b Department of Physical Therapy, I-Shou University, Kaohsiung; c Department of Emergency Medicine, College of Medicine, National Cheng Kung University, Tainan, Taiwan.

**Keywords:** right-sided native endocarditis, sternal abscess, paraspinal abscess, OSSA bacteremia, immunological dysregulation

## Abstract

**Introduction:**

Right-sided native endocarditis is a difficult case with fewer cardiac symptoms and fewer classic signs of cutaneous vascular lesions compared with left-sided endocarditis.

**Patient concerns:**

A 68-year-old Taiwanese man with a history of gouty arthritis, hyperlipidemia, and adrenal insufficiency presented to our ED and complained dyspnea and low back pain for 1 month.

**Diagnosis assessment:**

The PE showed bilateral crackles on chest auscultation and a palpable fluctuant mass over the anterior chest wall. The chest and abdominal CT scan showed multiple abscess formations involving pulmonary, sternal, and paraspinal areas. The TEE being performed and an oscillating mass over the anterior and septal leaflets of the tricuspid valve and moderate tricuspid regurgitation.

**Interventions:**

Only pharmacologic treatment without surgical interventions.

**Outcomes:**

Deceased, patient expired on day 4 after ED visit.

**Conclusion:**

This case arose as a sequela of staphylococcal endocarditis associated with persistent bacteremia and immunological dysregulation. The diagnosis of right-sided endocarditis is easily missing, multidisciplinary approach should be triggered as soon as possible, which might lead to a better outcome. Right-sided IE is still an important public health issue in southern Taiwan.

## 1 Introduction

Infective endocarditis (IE) remains an important clinical problem. It has high mortality (up to 40%), and its incidence has not significantly changed over the past 30 years.^[1,2]^ Although rheumatic heart disease remains the important predisposing factor for IE in developing countries,^[3]^ the rise of intravenous drug misuse, degenerative valve disease in the elderly, preceding valve surgery or vascular instrumentation, and nosocomial disease are the major changes in the incidence of IE.^[4]^

Endocarditis is caused by microbial infection of the endocardial surface or prosthetic material in the heart. Staphylococci, streptococci, enterococci, *Haemophilus*, *Actinobacillus*, *Cardiobacterium*, *Eikenella*, and *Kingella* (HACEK), are common pathogens in IE patients.^[1–4]^*Staphylococcus aureus* has surpassed viridans streptococci as the most common cause of IE in recent years. In addition, coagulase-negative staphylococci are the most important pathogens in early prosthetic-valve endocarditis and in native-valve endocarditis.^[3]^ The mitral and aortic valves are the most commonly infected valves. The tricuspid valve, although less affected than the mitral and aortic valves in IE, is more commonly involved in right-sided endocarditis.^[4]^

The mean annual incidence of IE in Taiwan from 1997 to 2002 was 7.6 per 100,000 inhabitants. During the surveyed period, the incidence was significantly increased from 4.8 per 100,000 persons in 1997 to 11 per 100,000 persons in 2002.^[5]^ Comparing with the trend in Western countries, which remained relatively constant and ranged from 1.5 to 6 episodes/100,000 person-years during the 1980s, the incidence of IE in Taiwan showed a little higher than those in Western countries.^[5,6]^ Right-sided endocarditis accounts for only 5% to 10% of cases of IE, most of these cases occur among injection drug users (IDUs). The incidence of IE in IDUs is approximately 1.5 to 20 per 1000 addicts per year.^[7]^ In Southern Taiwan, a higher incidence (11%) of IE caused by Methicillin-resistant *S aureus* (MRSA) in IDUs was reported.^[8]^

Clinical manifestations caused by IE are usually subtle and easily missed.^[9]^ The diagnosis of IE is made based upon a synthesis of clinical, microbiological, and echocardiographic findings.^[6]^ The modified Duke criteria^[10]^ are the standard criteria used to guide the diagnosis of IE. The two major criteria for a definite diagnosis of IE are positive blood cultures and evidence of endocardial vegetations on either a transthoracic (TTE) or transesophageal echocardiogram (TEE). There are also five minor criteria: persistent fever, the risk factors of an invasive procedure within 60 days, a prosthetic valve, current intravenous drug use, and the classic signs of Janeway lesions, Osler's nodes or Roth's spots (vascular and immunologic phenomena). However, their absence does not rule out the diagnosis of IE, and the risk factors should be highly suspected and antibiotic treatment empirically initiated.^[2,4]^ The identified new risk groups for IE necessitate an accelerated evaluation strategy for early diagnosis and early surgical intervention to decrease complications and death.^[4,6]^ Here, we report a case of isolated tricuspid valve IE with multiple abscess formations.

## 2 Case presentation

A 68-year-old Taiwanese man with a history of gouty arthritis, hyperlipidemia, and adrenal insufficiency presented in our ED after 1 month of dyspnea and low back pain. He had no history of recent surgery or illicit substances use; however, previous frequent intravenous and/or intramuscular drug injections over shoulders and buttocks for gout attacks by local clinicians were mentioned. The patient denied smoking history and had no other notable medical record except low dose prednisolone prescribed. He also denied any recent invasive procedures or surgeries. The patient had consulted another hospital for similar symptoms before this visit. He was admitted and given serial examinations—an abdominal computed tomography (CT) scan and a TTE—but only a sternal fracture with a closed perifocal hematoma was found. During admission, the patient was diagnosed with Oxacillin-sensitive *S aureus* (OSSA) bacteremia, but no vegetation was detected using cardiac imaging. He was discharged with an uneventful course on his first admission.

The patient's persistent dyspnea and low back pain worsened, and he returned to our ED again 2 weeks later. On presentation, the patient was afebrile and had a pulse rate of 132 beats/min, a respiratory rate of 20 breaths/min, and a blood pressure of 117/87 mm Hg. A physical examination showed bilateral crackles, a right-side wheeze on chest auscultation, and a palpable fluctuant, non-tender mass (3 × 3 cm) over the anterior chest wall. There was no heart murmur and no skin rash over the chest wall. The patient's abdomen was soft without hepatosplenomegaly. His lower legs were not swollen, and they had no skin rash. No visible erythematous changes over hand (Janeway erythema) nor painful spots over fingers (Osler's nodes) being detected. The patient's laboratory data revealed the following: White blood cell count = 17.2 × 10^9^/L (normal: 3.4–9.1 × 10^9^/L) with 76% polys and 16% bands; platelet count = 32 × 10^9^/L (normal: 138–353 × 10^9^/L); prothrombin time = 14.3 s (control: 9.4–12.5 s); C-reactive protein = 270.0 mg/L (normal: 0–8 mg/L); blood glucose = 259 mg/dL (normal: <140 mg/dL); blood urea nitrogen = 62 mg/dL (normal: 7–21 mg/dL); serum creatinine = 1.8 mg/dL (normal: 0.7–1.5 mg/dL); alanine aminotransferase = 58 U/L (normal: 0–54 U/L); lactate = 7.2 mmol/L (normal: 0.7–2.1 mmol/L); D-dimers (fibrin degradation product) > 3500 ng/mL; fibrinogen = 546.8 mg/dL (normal: 30–400 mg/dL). Arterial blood gas measured in the ED revealed severe metabolic acidosis (pH, 7.28; PaCO_2_, 19.2; PaO_2_, 226; HCO_3_^−^, 8.9; BE, −18, SaO_2_, 100%) under an FiO_2_ 1.0 concentration (non-rebreathing mask). No pyuria or hematuria was detected. An initial chest radiograph in the ED revealed no significant findings, and the electrocardiogram showed sinus tachycardia. A blood specimen was obtained for culturing and intravenous antibiotic agents (piperacillin and levofloxacin) were empirically initiated.

**Figure 1:**
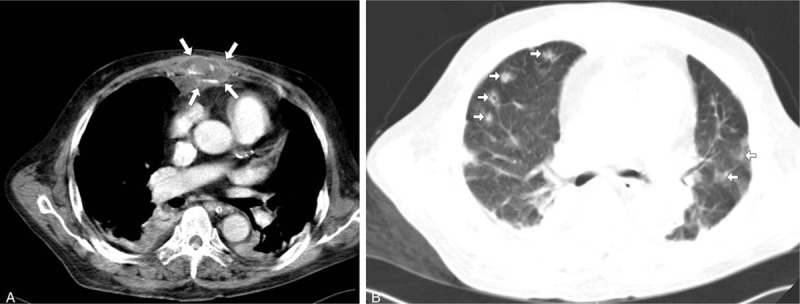
A. Contrast-enhanced spiral computed tomography scan (transverse section) of the chest shows a heterogeneous mass over the sternal area with enhanced periosteal infiltration (arrows). B. Multiple nodular opacities over bilateral lung parenchyma, which were compatible with septic embolic lesions (arrows), some of which are cavitated.

**Figure 2:**
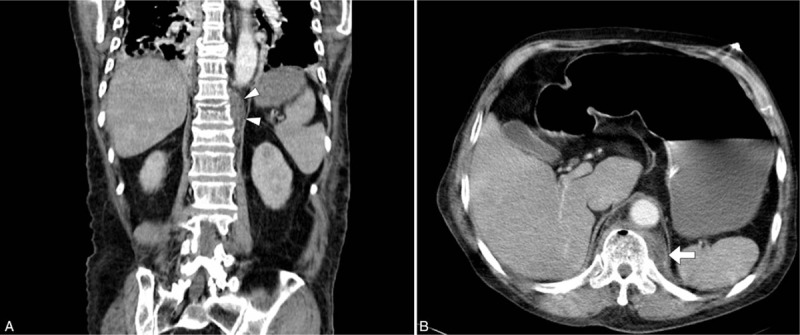
A. Computed tomography scan (coronal section) of the abdomen shows increasing paraspinal soft tissue enhancement and swelling along the left lumbar spine (from L_1_ to L_5_) (arrowheads). B. An anterior paraspinal abscess formation identified at the L_2_ compressed fracture with gas density is shown on the transverse section (arrow).

Because the patient's hemodynamic status and oxygen saturation rapidly deteriorated during observation in the ED, computed tomography of both chest and abdomen was arranged to detect a possible infection focus. A hypodense lesion with a peripherally enhanced wall and bone involvement was found in his sternal region (Fig. 1A), and multiple septic emboli lesions were found in his bilateral lung fields (Fig. 1B). Moreover, there was a paraspinal hypodense lesion with a peripherally enhanced wall and air-trapping over the T_11_ level. There was also one linear fracture line on T_11_ (Fig. 2A), and an L_2_ compressed fracture with a small anterior paraspinal abscess with gas density (Fig. 2B). CT-guided percutaneous drainage from the T_11_-T_12_ paraspinal space was done on day 2, and only minimal pus-like fluid was obtained for culture.

**Figure 3:**
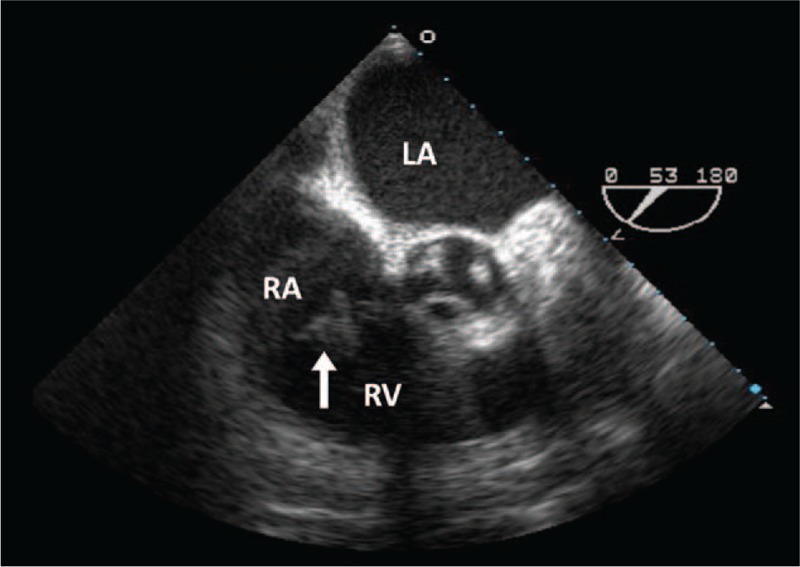
Transesophageal echocardiogram. A parasternal, short-axis view from the transesophageal echocardiogram shows a mobile mass consistent with vegetation adjacent to the tricuspid valve (white arrow). RA denotes right atrium, RV, right ventricle, and LA, left atrium.

Because of the septic pulmonary emboli, TEE was done on day 3; it showed an oscillating mass (1.56 cm in size) over the anterior and septal leaflets of the tricuspid valve and moderate tricuspid regurgitation (Fig. 3). The mitral, aortic, and pulmonary valves were intact, and there were no intracardiac shunts, either ASD (atrial septal defect) or PFO (perforation of foramen ovale) being found. Cardiovascular and chest surgeons were consulted immediately for emergency surgical intervention, but the family did not wish to pursue aggressive interventions and the patient eventually expired on day 4. OSSA was isolated from the blood culture and pus-like aspirate. The diagnosis was right-sided IE complicated with sternal abscesses, septic pulmonary emboli, and lumbar spondylodiscitis.

## 3 Discussion

Right-sided endocarditis demonstrates with fewer cardiac symptoms and fewer classic signs of cutaneous vascular lesions than shown in left-sided IE.^[7]^ Right-sided IE is often found in intravenous drug users complicated by septic lung embolization, but it is rarely presented with systemic emboli, as in our case. Although right-sided IE always has a better outcome than left-sided IE, persistent bacteremia with multiple abscesses, older age, and devastating pulmonary conditions lead to fatal results. Our patient had no history of pre-existing valvular diseases or drug addiction use; we therefore suspected that frequent injections for treatment of gouty arthritis might have caused a peripheral infection. It is noteworthy that the immunocompromised state caused by using steroids for adrenal insufficiency also contributes to an afebrile and devastating course. *S aureus*, a sufficiently virulent microorganism to infected valves, is the most common pathogen of right-sided native valve endocarditis, both in Western and Taiwanese populations.^[7,8]^ Although streptococcus species are the most important pathogens for community-acquired IE in Taiwan, staphylococcal species are the major pathogens (75%) of right-sided endocarditis.^[8,11]^*S aureus* is more likely to induce severe sepsis, persistent bacteremia, and multiple organ failure, all of which lead to higher mortality.^[12]^

Extracardiac or possibly intrapulmonary shunting^[13]^ might provide an unusual conduit for systemic spreading in right-sided IE. Moreover, massive bacterial loads from recurrent injections and immunological dysregulation might contribute to persistent bacteremia,^[7,14]^ which led to sternal, pulmonary, and paraspinal abscesses in our case. For patients with persistent OSSA bacteremia and multiple extracardiac manifestations, as shown in our case with multiple abscesses, TEE should be done early despite a negative transthoracic echocardiogram.^[1]^

About 5% of patients with IE are complicated with spondylodiscitis, which primarily involves the lumbar spine.^[15]^ Most of these patients, have left-sided endocarditis, and the major manifestations are back pain and fever.^[16]^ The risk factor of IE complicated with spondylodiscitis is predisposing heart disease, such as intracardiac device replacement.^[16]^ However, IE complicated with spondylodiscitis does not worsen the prognosis, but it does prolong hospitalization and increase the probability of surgical debridement.^[17]^ Actually, surgery should generally be avoided in right-sided IE, unless big vegetations (>20 mm), right heart failure induced by tricuspid valve regurgitation, or persistent bacteremia despite adequate antibiotic treatment develop.^[6]^ The goals of surgery are to eradicate the infection and achieve hemodynamic correction. With adequate strategies, the surgery can yield good results.^[17]^

In summary, the response of *S aureus* IE to antimicrobial therapy is slow, and many patients remain persistent bacteremia for weeks.^[18]^ Multiple visceral abscesses, as shown in our case, are frequently found in patients with prolonged fever associated with staphylococcal endocarditis. Traditionally, beta-lactamase-resistant penicillins, first generation cephalosporins, and aminoglycosides besides vancomycin for patients allergic to penicillins have been used as the main drugs in the treatment of staphylococcal endocarditis. Early consultation with a cardiac surgeon is still important and recommended in order to determine the best therapeutic approach.

## 4 Learning points

1. Right-sided infectious endocarditis with native tricuspid valve vegetation causes septic pulmonary emboli and presenting with systemic septic emboli is unusual, compared with left-sided endocarditis, and always associates with poor outcome.
2. Staphylococcal species, rather than streptococcus specie, are the major pathogens of right-sided endocarditis in southern Taiwan.
3. Early trigger a multidisciplinary approach involving microbiologists, cardiologists, and cardiothoracic surgeons, which might lead to a better outcome in these cases.

## Author contributions

**Conceptualization:** Chia-Chang Chuang.

**Data curation:** Tsung-Yu Chan, Chien-Liang Chen, Yao-Yi Huang, Chia-Chang Chuang.

**Formal analysis:** Chia-Chang Chuang.

**Methodology:** Chih-Chia Hsieh, Chien-Liang Chen, Yao-Yi Huang.

**Resources:** Chih-Chia Hsieh.

**Supervision:** Chia-Chang Chuang.

**Validation:** Yao-Yi Huang, Chia-Chang Chuang.

**Visualization:** Yao-Yi Huang.

**Writing – original draft:** Tsung-Yu Chan.

**Writing – review & editing:** Chih-Chia Hsieh, Yao-Yi Huang, Chia-Chang Chuang.

Chia-Chang Chuang orcid: 0000-0003-0481-2949.
